# Epilepsy Knowledge, Attitudes, and First‐Aid Practices Among Syrian University Students: A Cross‐Sectional Study

**DOI:** 10.1002/hsr2.73005

**Published:** 2026-08-09

**Authors:** Ahmad Al Darra, Fares Kahal, Saeed Kadri, Jonathan‐Raphaël Stetco, Mohamad Tharwat Afandi, André Torbey, Youssef Latifeh

**Affiliations:** ^1^ Faculty of Medicine Syrian Private University Damascus Syria; ^2^ Faculty of Medicine University of Sherbrooke Quebec Canada; ^3^ Faculty of Medicine Al Andalus University for Medical Sciences Tartus Syria; ^4^ Faculty of Medicine Damascus University Damascus Syria

**Keywords:** Attitudes, Epilepsy, First‐aid practices, Knowledge, Stigma, Syrian university students

## Abstract

**Background and Aims:**

Epilepsy affects about 50 million people worldwide, with stigma and misconceptions that contribute to social exclusion and psychological distress, sometimes more disabling than the physical symptoms. In Syria, epilepsy awareness among university students remains under‐studied, despite their role as future healthcare providers and community leaders. This study assessed epilepsy‐related knowledge, attitudes, and first‐aid practices among Syrian university students and identified associated factors.

**Methods:**

A cross‐sectional study was conducted including 1005 students from 24 Syrian universities using a structured questionnaire that covered demographics, epilepsy knowledge, first aid, and attitudes. Reliability was assessed using Cronbach's alpha. Data were analyzed using descriptive statistics, t‐tests, ANOVA, and logistic regression, with statistical significance set at *p* < 0.05.

**Results:**

High epilepsy knowledge was found in 59% of students, but misconceptions persisted. Although 63% showed good first‐aid knowledge, harmful practices were common. Attitudes were mixed, with 56% expressing positive views but significant reluctance toward close personal and professional relationships, potentially leading to social isolation, discrimination in employment and education, and reduced opportunities for marriage and community integration.

**Conclusions:**

Syrian students show moderate knowledge but persistent stigma and unsafe practices. Targeted education and first‐aid training are needed to reduce misconceptions and improve support for people with epilepsy.

## 1 Background

Epilepsy is one of the most prevalent neurological disorders globally, affecting approximately 50 million individuals worldwide. Nearly 80% of those with epilepsy reside in developing countries, where access to medical care and educational resources regarding the condition is often limited [1]. This condition is characterized by recurrent, unprovoked seizures resulting from abnormal electrical activity in the brain, leading to acute physical manifestations such as impaired consciousness, involuntary motor activity, and urinary incontinence [2]. Moreover, widespread misconceptions regarding the causes, treatment, and nature of epilepsy contribute to pervasive stigma, which often negatively affects the quality of life of people with epilepsy (PWE) [3]. Cultural and religious beliefs further shape these perceptions, with epilepsy in some settings being viewed as a mental illness or in extreme cases, a form of divine punishment [3]. These misconceptions and stigmatizing attitudes remain prevalent across diverse populations, as documented in numerous studies demonstrating that epilepsy‐related stigma is consistently associated with social exclusion and discrimination in areas like employment, education, and marriage. These factors ultimately contribute to poorer outcomes for PWE, often making the psychosocial impact of the condition exceed the burden of its clinical manifestations [4, 5, 6, 7, 8]. Furthermore, the limited understanding of this condition can lead to inappropriate and potentially harmful interventions during seizure episodes, as seen in studies from various countries [6, 9]. Unfortunately, gaps in knowledge are not limited to laypersons but extend to healthcare professionals and students in training, underscoring the importance of educational interventions not only for the general population but across the aforementioned groups to improve understanding and reduce stigmatization [10].

In Syria, epilepsy remains a largely under‐researched area, particularly concerning public awareness and attitudes toward the condition. University students, as future leaders and potential healthcare providers, represent a crucial demographic for understanding and shaping public perceptions of epilepsy. However, little is known about their level of knowledge regarding the condition, their attitudes toward people with epilepsy, and their ability to administer appropriate first aid during seizures. Given the broader regional trends of stigma and misunderstanding observed in neighboring countries, it is likely that similar challenges exist in Syria [6, 7].

Notably, epilepsy and its first aid are generally not included in the curricula of Syrian schools or university programs, except within medical faculties. This lack of formal education on the topic in non‐medical fields limits awareness and understanding among the broader student population. Moreover, the lack of awareness programs about epilepsy in Syria perpetuates misconceptions and stigma, hindering efforts to improve public knowledge and reduce discrimination.

This study aims to comprehensively assess the knowledge, attitudes, and first‐aid practices related to epilepsy among university students in Syria, and to identify the demographic and educational factors that influence these aspects. By addressing these critical gaps, the findings of this research could inform public health initiatives and educational programs designed to improve epilepsy awareness and reduce the stigma associated with the condition.

## 2 Methods

### 2.1 Research Design

A cross‐sectional study design was used to collect data from university students in Syria.

### 2.2 Study Population and Sampling

The target population consisted of university students enrolled at 24 Syrian universities, including 9 public universities (Damascus University, Aleppo University, Tishreen University, Al‐Baath University, Hama University, Al‐Furat University, University of Tartus, Higher Institute for Applied Sciences and Technology, and Syrian Virtual University) and 15 private universities (Syrian Private University, International University for Science and Technology, Arab International University, Al‐Hawash Private University, University of Kalamoon, Wadi International University, Al‐Sham Private University, Al‐Jazeera Private University, Al‐Qalamoun Private University, Cordoba Private University, Ebla Private University, Mamoun University for Science and Technology, International University for Medical Sciences, Al‐Andalus Private University, and Manara University).

All currently enrolled students were eligible, with no restrictions by age, sex, or academic discipline. Convenience sampling was used, with representation from medical and non‐medical fields. Based on an estimated population of 600,000 students, a minimum sample size of 384 was calculated (95% confidence level, 5% margin of error, 50% population proportion). A total of 1005 students participated, significantly exceeding this requirement and ensuring robust statistical power.

### 2.3 Questionnaire Design

A structured questionnaire assessed knowledge, attitudes, and first‐aid practices related to epilepsy. It consisted of four sections: demographics, epilepsy knowledge, first‐aid during seizures, and attitudes toward people with epilepsy. This questionnaire was based on validated questionnaires: the first‐aid section adapted from Abbasi‐Kangevari et al., and the knowledge and attitude sections from Abuhamdah et al. [4, 11] The drug therapy question from AbuHamdah et al. was replaced with a question adapted from Shihata et al., as the original item required in‐depth knowledge of epilepsy treatment beyond what would be expected from students in non‐medical fields. The replacement question was broader in scope and more appropriate for students from diverse academic disciplines [11, 12].

Pilot testing was conducted with 50 students to assess clarity and length, and revisions were made accordingly. Internal consistency was acceptable, with Cronbach's alpha values ranging from 0.63 to 0.68.

#### 2.3.1 Demographic Information

Collected data included age, sex, university, academic year, field of study, parents' education, and economic status to explore factors influencing awareness and perception about epilepsy.

#### 2.3.2 Knowledge of Epilepsy

This section included multiple‐choice questions on causes, symptoms, and management of epilepsy. For example, questions that addressed the occurrence of epilepsy and the recognition of seizure attacks [11, 12]. A 15‐item scale was used, with one point per correct answer (maximum score 15). Scores were categorized as low (below the mean) and high (above the mean).

#### 2.3.3 First‐Aid Practices

This section assessed the knowledge of seizure first‐aid measures, including appropriate and inappropriate actions. For example, questions that addressed whether participants would place the person in a safe position, avoid restraining movements, and refrain from inserting objects into the mouth during a seizure [4]. A 14‐item scale was used, with one point awarded for performing appropriate actions or avoiding harmful ones (maximum score 14). Scores were categorized as low (below the mean) and high (above the mean).

#### 2.3.4 Attitudes Towards Epilepsy

Attitudes were assessed using a four‐point Likert scale, where 1 = strongly disagree, 2 = disagree, 3 = agree, and 4 = strongly agree, with a maximum possible score of 48. The scale did not include a neutral midpoint. This design was used to minimize neutral responses and encourage participants to express a clear positive or negative attitude, thereby reducing central tendency bias. Negatively worded items (items 7 and 11) were reverse‐scored. This section assessed stigma toward people with epilepsy. For example, questions that included willingness to work with or accept people with epilepsy as marriage partners, and views on whether they should attend school [11].

### 2.4 Data Collection

Data were collected using online (Google Forms) and paper‐based surveys distributed through university platforms, social media, and on campus. Participants were briefed on the study objectives and provided informed consent before independently completing the survey without author interference. To eliminate duplicate responses, the dataset was screened and responses with duplicate demographic information were removed during data analysis. The level of control over participant responses was comparable between the online and paper‐based questionnaires, as both were completed independently without direct monitoring by the authors.

### 2.5 Statistical Analysis

Data were analyzed using IBM SPSS Statistics version 26. Descriptive statistics were calculated. Independent sample t‐tests and one‐way ANOVA assessed relationships between demographics and knowledge, attitudes, and first‐aid scores. Binary logistic regression analysis was conducted to identify predictors of good knowledge, high first‐aid knowledge, and positive attitudes. Cut‐off values were defined as scores above the mean to assess relative participant performance: knowledge > 7.43 (SD 3.03), attitude > 35.07 (SD 4.78), and first‐aid > 10.96 (SD 1.91). Statistical significance was set at *p* < 0.05.

## 3 Results

### 3.1 Participants Characteristics

**Figure 1:**
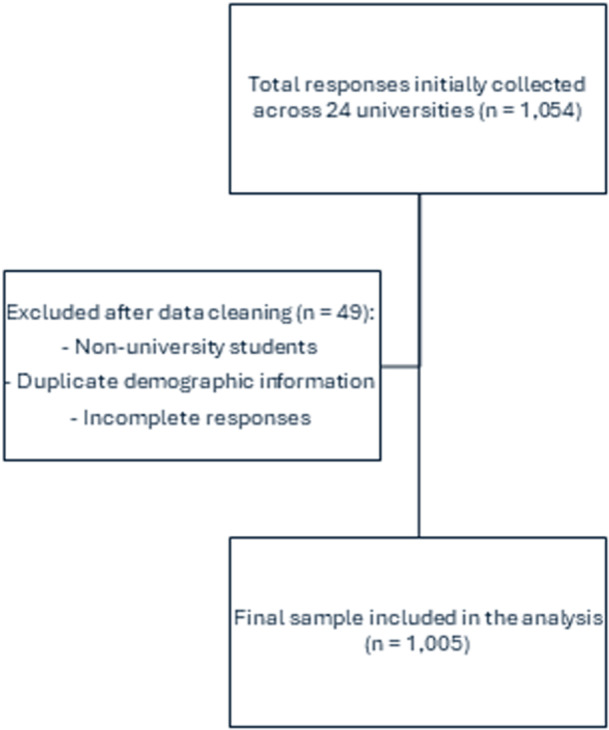
Participant flow diagram illustrating the selection process and data cleaning.

Initially, 1054 responses were collected. After data cleaning and the removal of 49 incomplete or duplicate responses, a total of 1005 participants were included in the final analysis (Figure 1), with a female majority (62%). The mean age was 22.27 years (± 3.47). Among the participants, 60% were enrolled in public universities, and 54% were in medical‐related fields. Furthermore, 52% were in the final 3 years of their university education. Also, 51% reported “good” monthly income. As well as 89% of the participants indicated hearing of or reading about epilepsy. Moreover, 40% personally knew someone with the condition, and 45% had witnessed an epileptic seizure (Table 1).

**Table 1:** Characteristics of the study participants.

Age	
Mean (± SD)	22.27 (± 3.47)
**Gender *N* (%)**	
Male	386 [38]
Female	619 [62]
**Residency**	
Home	930 [93]
University accommodation	60 [6]
Other	15 [1]
**University**	
Public	608 [60]
Private	397 [40]
**Faculty**	
Medical	547 [54]
Non‐medical	458 [46]
**Year**	
First‐year	156 [13]
Second year	158 [13]
Third year	175 [14]
Fourth year	197 [15]
Fifth year	197 [15]
Sixth year	122 [12]
**Parents education**	
No formal education	34 [3]
School level	266 [16]
University level	598 [60]
Higher education	107 [11]
**Parents involved in the medical field**	
Yes	153 [17]
No	852 [85]
**Economic status**	
Low	50 [5]
Moderate	200 [15]
Good	515 [51]
Excellent	240 [18]
**Have you ever heard or read about epilepsy?**	
Yes	897 [89]
No	108 [11]
**Do you know or have you ever known anyone who had epilepsy?**	
Yes	400 [40]
No	605 [60]
**Have you ever seen anyone having an epileptic seizure?**	
Yes	452 [45]
No	553 [55]

### 3.2 Knowledge About Epilepsy

**Figure 2:**
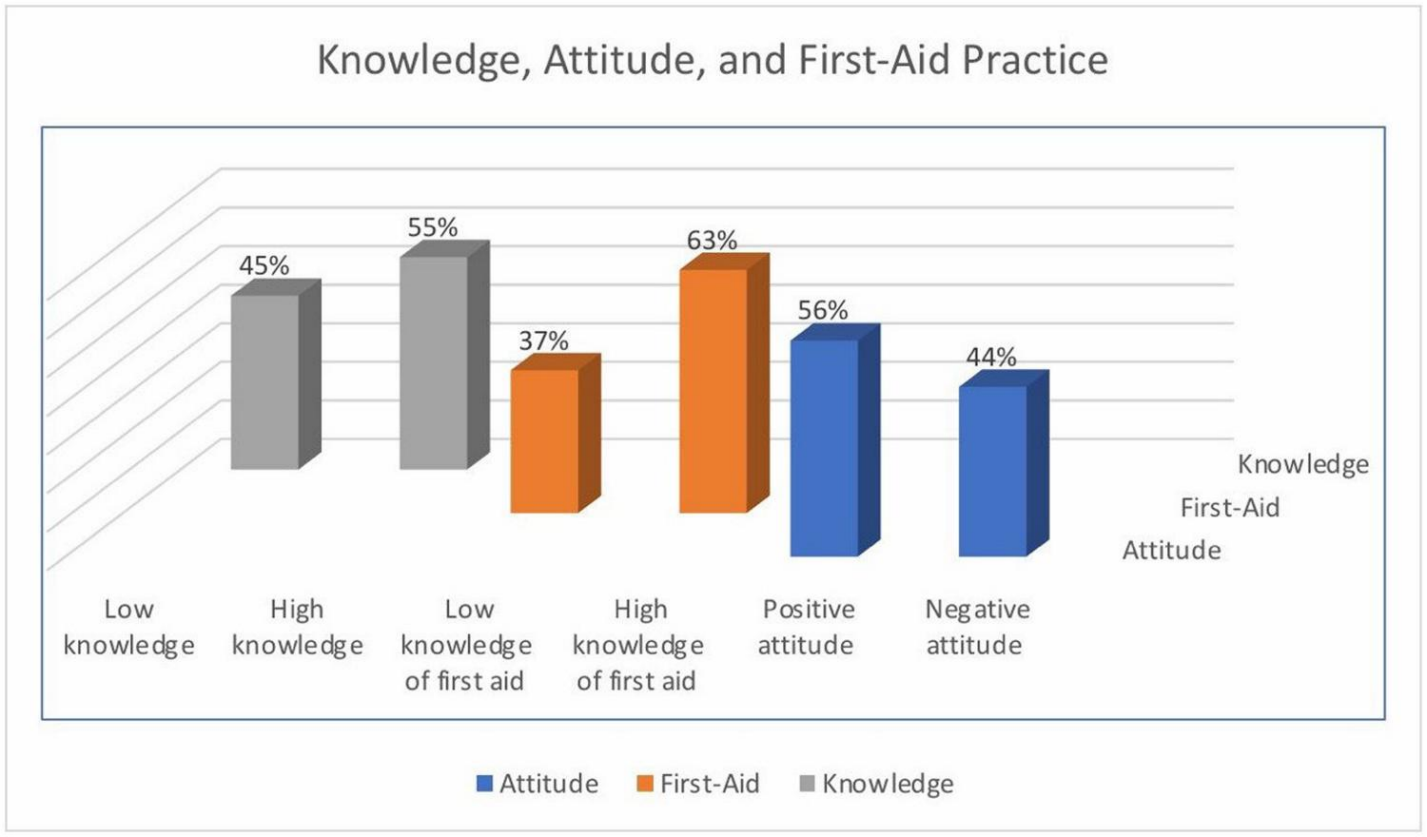
Proportion of individuals categorized by their knowledge of epilepsy, first‐aid comprehension, and overall attitudes.

The participants exhibited varied levels of knowledge about epilepsy, with correct responses ranging between 22% and 73%. The average score was 7.43 (± 3.03) out of 15, with 55% demonstrating a high level of knowledge (Figure 2). Fewer than 25% were aware of the accurate incidence rate of the condition. Awareness of its causes varied, with 17% identifying stroke and 40% recognizing inherited diseases as potential causes. Understanding of epileptic attacks was also inconsistent, with 61% recognizing loss of consciousness, and only 27% identifying memory disturbances. Regarding management, 74% recognized the importance of seeking a doctor's referral, while 22% mentioned neurosurgery as a treatment option. Recognition of seizure symptoms varied between 36% for eye‐rolling and 73% for jerking movements of the arms and legs. Additionally, 92% of participants correctly responded “no” to the question regarding whether epilepsy is contagious (Table 2).

**Table 2:** Participants' responses to knowledge items.

Epilepsy occurs in	*N* (%)
One in every 100 people*	218 [19]
One in every 1000 people	389 [39]
One in every 10.000 people	282 [28]
One in every 50.000 people	90 [9]
One in every 1000.000 people	26 [3]
**What do you think causes epilepsy? (more than one answer could be chosen)**	
Accident*	383 [31]
Inherited disease*	489 [40]
Birth defects*	268 [19]
Insanity or other mental illness	564 [46]
Brain tumors*	414 [34]
Stroke*	208 [14]
Don't know	95 [8]
**What do you think an epileptic attack is? (more than one answer could be chosen)**	
A loss of consciousness*	740 [61]
An episode of behavioral change*	529 [43]
A period of memory disturbance*	325 [20]
Don't know	60 [5]
**What do you think about drug therapy for epilepsy? (more than one answer could be chosen)**	
Needle therapy	152 [21]
Neurosurgery*	266 [19]
Herbal medicine	68 [6]
Doctor referral*	908 [74]
**What are the signs and symptoms of epileptic seizures you know of? (more than one answer could be chosen)**	
Falling down*	764 [63]
Rolling of the eyes*	439 [36]
Foaming of the mouth*	634 [52]
Uncontrolled jerking movement of the arms and legs*	889 [73]
**Is epilepsy contagious?**	
Yes	9 [1]
No*	926 [92]
Do not know	70 [7]
**Is epilepsy a dangerous condition?**	
Yes*	680 [68]
No	197 [15]
Do not know	128 [12]
**Overall knowledge**	
Low knowledge (< 9)	451 [45]
High knowledge (≥ 9)	554 [55]
**Overall knowledge score**	
Mean (± SD)	9.03 (± 3.18)
Lowest value	2
Highest value	17

* The correct answer(s).

### 3.3 First‐Aid Knowledge

The average first‐aid knowledge score was 10.96 (± 1.91) out of 14, with 63% achieving a high score (Figure 2). Most participants (97%) were aware of the importance of staying with the person until an ambulance arrives, and 93% of the need to clear up hazards and loosen clothing. Preventing falls (83%) and rolling the person onto their side (74%) were also commonly identified. However, harmful actions were prevalent, such as attempting to open the patient's mouth (43%) or sprinkling water (41%). (Table 3).

**Table 3:** Awareness about first‐aid measures at time of seizures.

Helpful measures	Yes *N* (%)	No *N* (%)
Staying with them until ambulance arrives	978 [97]	27 [3]
Clearing the area of dangerous objects	932 [93]	73 [7]
Preventing them from falling	839 [83]	166 [14]
Loosening clothing around the person's neck	935 [93]	70 [7]
Taking them to hospital right after seizures	751 [75]	254 [22]
Putting a pillow under their neck	726 [72]	279 [28]
Rolling them carefully on their side	745 [74]	260 [16]
**Potentially harmful measures**		
Attempting to open the mouth to put something between jaws	435 [43]	570 [57]
Trying to restrain the person	481 [48]	524 [52]
Shouting and moving to wake them up	177 [23]	828 [82]
Sprinkling water on face to awaken them	411 [41]	594 [59]
Administering drugs orally	127 [21]	878 [87]
Pouring water with sugar into their mouth	140 [24]	865 [86]
Forcing water down their throat	148 [17]	857 [85]
**Overall first‐aid score**		
Mean (± SD)	10.96 (± 1.91)	
Lowest value	2	
Highest value	14	
Low knowledge of first aid	375 [37]	
High knowledge of first aid	630 [63]	

### 3.4 Attitude Towards Epilepsy

The average attitude score was 35.07 (± 4.78) out of 48, with 56% showing positive attitudes (Figure 2). Attitudes varied significantly across different scenarios. For instance, 97% of participants were comfortable shaking hands with people with epilepsy, but only 29% were willing to employ them as household assistants. (Table 4).

**Table 4:** Participants' responses to attitude items.

Variable	Strongly disagree *N* (%)	Disagree *N* (%)	Agree *N* (%)	Strongly agree *N* (%)
Agree to work with people with epilepsy	64 [6]	166 [14]	639 [64]	136 [24]
Agree to have close relation with people with epilepsy	38 [4]	172 [14]	651 [65]	144 [24]
Agree to live together with people with epilepsy	58 [6]	219 [19]	605 [60]	123 [12]
people with epilepsy should not be isolated	57 [6]	73 [7]	450 [45]	425 [42]
people with epilepsy can manage their family	73 [7]	195 [25]	535 [53]	202 [15]
Agree to shake hands with people with epilepsy	13 [1]	24 [2]	399 [40]	569 [57]
Keep child from contacting people with epilepsy	466 [46]	334 [33]	169 [14]	36 [4]
Agree to recruit people with epilepsy as a household assistant	280 [28]	433 [43]	232 [26]	60 [6]
Agree with family member marrying a person with epilepsy	144 [24]	385 [38]	390 [39]	86 [9]
Epilepsy is a treatable disease	48 [5]	316 [31]	523 [52]	118 [12]
People with epilepsy should not learn in schools	605 [60]	177 [23]	116 [12]	107 [11]
People with epilepsy can lead a healthy lifestyle	34 [3]	60 [6]	540 [54]	317 [37]
**Overall attitude score**	35.07 (± 4.78)			
Lowest value	20			
Highest value	48			
Positive attitude	561 [56]			
Negative attitude	444 [44]			

### 3.5 Relationship Between Participants' Characteristics and Total Knowledge Score, First‐Aid Score and Attitude Score

Epilepsy knowledge, first‐aid understanding, and overall attitudes varied significantly based on gender, academic background, and personal exposure. Females (9.23 ± 3.00) demonstrated better knowledge than males (8.70 ± 3.41, *p* = 0.01), while medical students (9.86 ± 3.13) and those from private universities (9.44 ± 3.13) outperformed non‐medical students (8.04 ± 2.93, *p* < 0.001) and those from public universities (8.76 ± 3.18, *p* = 0.001). Total first‐aid knowledge and attitudes scores were also higher among private university students (11.20 ± 1.88, *p* = 0.001; 35.94 ± 5.10, *p* < 0.001) and medical students (11.28 ± 1.87, *p* < 0.001; 35.89 ± 4.56, *p* < 0.001). Scores improved consistently from first‐year to sixth‐year students (first‐aid: 10.47 ± 1.93 to 11.54 ± 1.72; attitudes: 34.35 ± 4.68 to 36.81 ± 4.36, *p* < 0.001). (Table 5).

**Table 5:** Relationship between participant's characteristics and total knowledge score, first‐aid score and attitude score.

Variables	Total knowledge score mean (±SD)	*p* value	Overall first‐aid score mean (±SD)	*p* value	Overall attitude score mean (±SD)	*p* value
**Gender**		0.01*		0.58		0.21
Male	8.70 (± 3.41)		10.92 (± 1.93)		34.83 (± 4.87)	
Female	9.23 (± 3.00)		10.99 (± 1.91)		35.22 (± 4.72)	
**Residency**		0.92		0.62		0.07
Home	9.04 (± 3.18)		10.98 (± 1.90)		35.19 (± 4.73)	
University accommodation	8.95 (± 3.35)		10.81 (± 2.11)		33.7 (± 5.43)	
Other	8.66 (± 2.41)		10.66 (± 1.79)		33.33 (± 4.32)	
**University**		0.001*		0.001*		<0.001*
Public	8.76 (± 3.18)		10.80 (± 1.92)		34.50 (± 4.48)	
Private	9.44 (± 3.13)		11.20 (± 1.88)		35.94 (± 5.10)	
**Faculty**		<0.001*		<0.001*		<0.001*
Medical	9.86 (± 3.13)		11.28 (± 1.87)		35.89 (± 4.56)	
Non‐medical	8.04 (± 2.93)		10.58 (± 1.90)		34.10 (± 4.86)	
**Year**		<0.001*		<0.001*		<0.001*
First‐year	7.49 (± 2.57)		10.47 (± 1.93)		34.35 (± 4.68)	
Second‐year	7.99 (± 3.02)		10.51 (± 2.01)		33.92 (± 4.91)	
Third‐year	8.24 (± 2.90)		10.51 (± 2.00)		34.26 (± 5.21)	
Fourth‐year	9.34 (± 2.98)		11.26 (± 1.66)		35.10 (± 4.62)	
Fifth‐year	10.13 (± 2.95)		11.46 (± 1.83)		36.19 (± 4.24)	
Sixth‐year	11.21 (± 3.23)		11.54 (± 1.72)		36.81 (± 4.36)	
**Parents education**		<0.001*		0.02*		<0.001*
No formal education	8.67 (± 2.45)		10.61 (± 2.01)		34.17 (± 5.38)	
School level	8.36 (± 3.20)		10.72 (± 1.88)		34.19 (± 5.17)	
University level	9.11 (± 3.07)		11.03 (± 1.92)		35.25 (± 4.61)	
Higher education	10.37 (± 3.43)		11.28 (± 1.88)		36.55 (± 4.07)	
**Parents involved in the medical field**		0.39		0.93		0.48
Yes	9.25 (± 3.46)		10.95 (± 2.00)		35.35 (± 5.44)	
No	8.99 (± 3.12)		10.96 (± 1.90)		35.02 (± 4.65)	
**Economic status**		0.11		0.12		0.06
Low	9.06 (± 3.43)		10.24 (± 2.24)		33.4 (± 4.50)	
Moderate	8.61 (± 3.15)		10.93 (± 1.78)		34.70 (± 4.70)	
Good	9.23 (± 3.04)		11.02 (± 1.92)		35.17 (± 4.62)	
Excellent	8.95 (± 3.39)		11.02 (± 1.91)		35.52 (± 5.16)	
**Have you ever heard or read about epilepsy?**		<0.001*		<0.001*		<0.001*
Yes	9.34 (± 3.11)		11.05 (± 1.90)		35.47 (± 4.63)	
No	6.49 (± 2.55)		10.23 (± 1.91)		31.74 (± 4.76)	
**Do you know or have you ever known anyone who had epilepsy?**		0.008*		0.31		<0.001*
Yes	9.36 (± 3.18)		10.89 (± 1.98)		35.94 (± 4.92)	
No	8.81 (± 3.16)		11.01 (± 1.87)		34.50 (± 4.60)	
**Have you ever seen anyone having an epileptic seizure?**		0.04*		0.72		0.004*
Yes	9.25 (± 3.11)		10.99 (± 1.94)		35.55 (± 4.95)	
No	8.85 (± 3.22)		10.94 (± 1.89)		34.68 (± 4.61)	

### 3.6 Predictors of Good Knowledge, First‐Aid and a Positive Attitude

Participants demonstrated a high level of epilepsy knowledge (mean: 9.03/17, 55%), a positive attitude (mean: 35.07/48, 56%), and strong first‐aid knowledge (mean: 10.96/14, 63%). Binary logistic regression identified factors influencing knowledge, attitude, and first‐aid levels towards epilepsy. Age was positively associated with knowledge (*p* = 0.006). Females scored higher in knowledge than males (OR: 1.43, *p* = 0.001). Medical students outperformed non‐medical peers in knowledge (OR: 2.51, *p* < 0.001), attitude (OR: 1.70, *p* < 0.001), and first‐aid (OR: 1.74, *p* < 0.001). Awareness of epilepsy enhanced knowledge (OR: 3.23, *p* < 0.001) and attitude (OR: 2.92, *p* < 0.001). Senior students had better scores in knowledge (OR: 2.43, *p* < 0.001), attitude (OR: 1.54, *p* = 0.001), and first‐aid (OR: 2.37, *p* < 0.001). Knowing someone with epilepsy improved attitudes (OR: 1.52, *p* = 0.002). (Table 6).

**Table 6:** Factors affecting participants' knowledge, attitude and first‐aid towards epilepsy positively.

Variables	Knowledge level		Attitude level		First‐aid level	
	Odds ratio (95%CI)	*p* value	Odds ratio (95%CI)	*p* value	Odds ratio (95%CI)	*p* value
**Age**	1.06 (1.02–1.12)	0.006*				
**Gender**						
Male	Ref					
Female	1.43 (1.07–1.91)	0.01*				
**Faculty**						
Non‐medical	Ref	Ref	Ref			
Medical	2.51 (1.89–3.33)	<0.001*	1.70 (1.31–2.22)	<0.001*	1.74 (1.33–2.27)	<0.001*
**Have you ever heard or read about epilepsy?**						
No	Ref					
Yes	4.14 (2.48–6.92)	<0.001*	2.92 (1.86–4.60)	<0.001*		
**Year of study**						
First three years	Ref	Ref	Ref			
Last three years	2.46 (1.79–3.39)	<0.001*	1.54 (1.19–2.01)	0.001*	2.37 (1.81–3.11)	<0.001*
**Do you know or have you ever known anyone who had epilepsy?**						
No			Ref			
Yes			1.52 (1.16–1.98)	0.002*		

## 4 Discussion

Epilepsy remains a significant global health concern, affecting over 50 million people and presenting substantial neurobiological, cognitive, and psychosocial challenges. Understanding public knowledge, attitudes, and first‐aid practices is crucial in reducing stigma and improving care for peoeple with epilepsy [3]. The significance of these findings is heightened in the Syrian setting, where prolonged healthcare system pressure and inadequate public health education campaigns may increase epilepsy‐related misconceptions and stigma [21]. To the best of our knowledge, this is the first study in Syria to comprehensively assess university students' knowledge, attitudes, and awareness of appropriate first‐aid responses to epileptic seizures.

In our study, a 55% of participants demonstrated a strong understanding of epilepsy, though there were misconceptions about its causes. Specifically, 46% of participants mistakenly believed epilepsy to be a psychological disorder, akin to insanity or mental illness (Table 2). A similar misconception was reported in a comparable Jordanian study, where 40.8% of participants held this belief [11]. Although epilepsy is associated with various psychiatric conditions, including depression, anxiety disorders, psychosis, and autism spectrum disorders, these associations do not imply that epilepsy itself is a psychological disorder [24].

Although 63% of respondents showed strong first‐aid knowledge for seizures, improper practices were common (Table 3). These included attempting to insert objects into the mouth (43%), restraining the person (48%), and sprinkling water on the face to awaken them (41%). These actions can lead to serious consequences, such as broken teeth, jaw injuries, or even fractures [17]. While fewer participants considered other harmful measures, like shouting or moving the person to wake them up (18%), administering oral drugs (13%), pouring sugar water into the mouth (14%), or forcing water down the throat (15%). These actions pose significant risks, including choking or aspiration [17].

Moreover, 56% of participants expressed positive attitudes toward people with epilepsy, while 38% opposed marriage to people with the condition and 14% strongly opposing it (Table 4). Similar studies conducted in diverse populations have also reported a high percentage of individuals unwilling to marry someone with epilepsy, highlighting that this stigma is not isolated but rather a widespread issue across diverse cultural and geographical contexts [5, 12, 13, 14]. Moreover, a significant percentage of our study participants expressed reluctance to employ people with epilepsy as household assistants, with 43% disagreeing and 28% strongly disagreeing, a trend similarly observed in a study from Jordan, where 45% held the same view [11]. The observed disparity between the relatively good knowledge levels and the specific less favorable attitudes observed in our study may indicate deeply rooted cultural and social beliefs that cannot be easily changed by factual knowledge alone [3].

Our findings suggest that knowledge levels of epilepsy, first aid, and attitudes toward people with the condition may be influenced by various factors (Table 6). Demographic factors, particularly age and gender, were significantly associated with epilepsy knowledge. Older participants were more likely to demonstrate a higher level of knowledge about epilepsy (OR = 1.06), suggesting that cumulative life experience and greater exposure to health‐related information over time may contribute to improved understanding of the condition. Female participants were also significantly more likely to have better epilepsy knowledge than male participants (OR = 1.43). This finding is consistent with previous studies that have identified female gender as a significant predictor of greater epilepsy‐related knowledge [11]. Furthermore, this observation is supported by the broader health literacy literature, which has shown that women may generally exhibit higher levels of health literacy and a better understanding of medical information than men [23]. Being aware of epilepsy significantly enhanced participants' knowledge (OR = 4.14) and attitudes (OR = 2.92). Awareness of such conditions may encourage individuals to learn more about them, ultimately enhancing their overall knowledge and attitudes toward them. Other similar studies have shown that a lack of overall knowledge about epilepsy often leads to negative attitudes, which can perpetuate stigma and discrimination against those living with the condition [15, 25]. For instance, a study in Egypt found that knowledge about the etiology of epilepsy serves as a protective factor against negative attitudes, suggesting that increased awareness can mitigate stigma [27].

Our study found that medical students were more than twice as likely to have better epilepsy knowledge (OR = 2.51), exhibit more positive attitudes (OR = 1.70), and demonstrate stronger first‐aid knowledge (OR = 1.74) than non‐medical students. A study conducted in Turkey also found that students in medical fields had a better knowledge of epilepsy than those in non‐medical disciplines [8]. This is likely due to medical students' greater exposure to patients with epilepsy and their education in neurology, both of which enhance their knowledge and understanding of epilepsy [19]. A study conducted by Ibor et al. found a positive correlation between educational attainment and epilepsy knowledge [26]. Similarly, Khanal et al. reported that teachers with college or university degrees demonstrated significantly better knowledge about epilepsy compared to their less‐educated peers [18]. This demonstrates that higher levels of education are associated with improved attitudes and knowledge of epilepsy. A similar trend was observed in our study, as senior students in the last 3 years of university had higher odds of possessing a high level of knowledge about epilepsy (OR = 2.46) and its first aid (OR = 2.37), as well as demonstrating better attitudes (OR = 1.54) compared to junior students in the first 3 years of university.

Moreover, our research demonstrated that personally knowing someone with epilepsy was associated with a more positive attitude toward the condition (OR: 1.52) (Table 6), which may be attributed to increased empathy. This finding aligns with a similar study in Jordan and two studies conducted in Germany [11, 16, 22].

To address the critical gaps identified in our study, a comprehensive educational intervention is essential. This intervention should aim to enhance understanding, correct misconceptions, and promote supportive attitudes toward people with epilepsy. Supporting this need, educational intervention studies conducted in Turkey have also demonstrated significant improvements in epilepsy‐related knowledge, attitudes, and first‐aid practices among participants [20]. Launching awareness campaigns on campus and through social media platforms can help reach a broader audience by focusing on dispelling myths about epilepsy, highlighting the importance of proper first‐aid measures, and promoting empathy and support for people with epilepsy.

Regular first‐aid training workshops should be conducted for students, emphasizing the correct management of epileptic seizures. These workshops should be interactive, allowing students to practice safe techniques and understand the risks associated with common misconceptions, such as inserting objects into the mouth or restraining the person. Collaboration with local health organizations and epilepsy associations can provide expert‐led sessions and resources, facilitating the development of educational materials tailored to the cultural context of Syria.

Implementing a system to evaluate the effectiveness of these interventions through pre‐ and post‐intervention surveys is important. Collecting feedback from participants will help continuously improve the educational content and delivery methods. Future research should focus on exploring epilepsy knowledge and attitudes among different populations across Syria, aiding in the creation of targeted interventions that improve understanding, reduce stigma, and enhance support for individuals with epilepsy.

## 5 Conclusion

In conclusion, our study found that while many participants had a good understanding of epilepsy, misconceptions about its causes and harmful first‐aid practices, like inserting objects into the mouth or restraining the person, persisted. Attitudes were mixed, with significant reluctance toward personal or professional interactions with epilepsy patients, reflecting widespread stigma. Factors associated with higher knowledge levels included age, the belief in epilepsy as a serious condition, female gender, enrollment in health‐related faculties, awareness of epilepsy, and being in the senior years of university. Positive attitudes toward epilepsy were more likely in individuals from health‐related faculties, those aware of epilepsy, senior university students, and knowing someone with epilepsy. Predictors of higher scores in first‐aid management included health‐related university fields and being in the senior years of university.

To address the gaps in epilepsy knowledge, misconceptions about seizure management, and the negative attitudes toward individuals with epilepsy, targeted interventions are needed. These should include awareness campaigns to correct misunderstandings, first‐aid training to improve seizure response, and partnerships with health organizations for comprehensive education. Continuous evaluation of these initiatives will help refine and enhance their effectiveness. Future research should focus on epilepsy knowledge and attitudes across Syria to create tailored interventions that reduce stigma and provide better support for those affected.

## 6 Limitations

This study has several limitations that should be considered. The use of convenience sampling may limit the generalizability of the findings, as the sample may not accurately represent all Syrian university students and could introduce selection bias. While specific data on the proportion of non‐medical students who had completed epilepsy‐related courses were not collected, the observed differences between medical and non‐medical students were consistent with expectations, with medical students demonstrating significantly higher levels of knowledge. Therefore, any potential influence of epilepsy‐related education among non‐medical students is unlikely to have substantially affected the overall findings.

The cross‐sectional design captures data at a single point in time, preventing the assessment of temporal changes or causal relationships. Additionally, the reliance on self‐reported data may have introduced response bias, including the possibility of socially desirable responses. Although duplicate responses were screened and removed during data analysis, the use of both online and paper‐based questionnaires distributed through multiple recruitment channels may have introduced additional selection bias. Furthermore, as questionnaires were completed independently without direct supervision, some participants may have consulted external sources while answering knowledge‐related questions, potentially influencing the accuracy of the measured knowledge levels.

The study's focus on university students may limit the applicability of the findings to the broader Syrian population, particularly individuals without access to higher education. In addition, cultural and regional factors that may influence knowledge and attitudes toward epilepsy were not explored in depth. Future studies should address these limitations to provide a more comprehensive understanding of epilepsy‐related knowledge and attitudes and to inform the development of targeted educational interventions.

## 7 Approvals

All authors have read and approved the final version of the article. Ahmad Al Darra had full access to all of the data in this study and takes complete responsibility for the integrity of the data and the accuracy of the data analysis.

Ahmad Al Darra and Fares Kahal affirm that this article is an honest, accurate, and transparent account of the study being reported; that no important aspects of the study have been omitted; and that any discrepancies from the study as planned (and, if relevant, registered) have been explained.

## Author Contributions

**Ahmad Al Darra:** conceptualization, methodology, investigation, validation, visualization, writing – review and editing, writing – original draft, project administration, resources, software, funding acquisition. **Fares Kahal:** conceptualization, methodology, investigation, validation, resources, project administration, writing – review and editing, writing – original draft, visualization, software. **Saeed Kadri:** formal analysis, data curation, writing – original draft, writing – review and editing. **Jonathan‐Raphaël Stetco:** writing – original draft, writing – review and editing. **Mohamad Tharwat Afandi:** data curation. **André Torbey:** supervision. **Youssef Latifeh:** supervision.

## Funding

The authors have nothing to report.

## Ethics Statement

The study protocol received a thorough review and approval from the Syrian Private University Ethical Review Board, ensuring compliance with ethical research standards. The committee fully endorsed the study without providing an approval number. Participation was entirely voluntary, with informed consent obtained from all participants prior to their involvement. Confidentiality and anonymity were maintained throughout the study, with data securely stored and accessed only by authorized personnel. The study aligns with the Declaration of Helsinki.

## Conflicts of Interest

The authors declare no conflicts of interest.

## Data Availability

All materials used in this study are owned by the authors, except for the questionnaires, which were adapted from open‐access studies and are properly referenced in the Materials section. All figures and tables are owned by the authors. No additional permissions were required for their use. The datasets used and/or analyzed during the current study are available upon reasonable request to the corresponding author.
